# Supplementary material for: Causal Relationship between Aging and Anorexia Nervosa: A White-Matter-Microstructure-Mediated Mendelian Randomization Analysis
Source: Biomedicines. 2024 Aug 16;12(8):1874. doi: 10.3390/biomedicines12081874 (PMC11351342; doi:10.3390/biomedicines12081874)
Supplement: Supplementary file 1 [file biomedicines-12-01874-s001.zip › Supplementary Figure/Figure S9/Leaveoneout plot of MR analysis for ubm-b-1520 to AN.pdf]

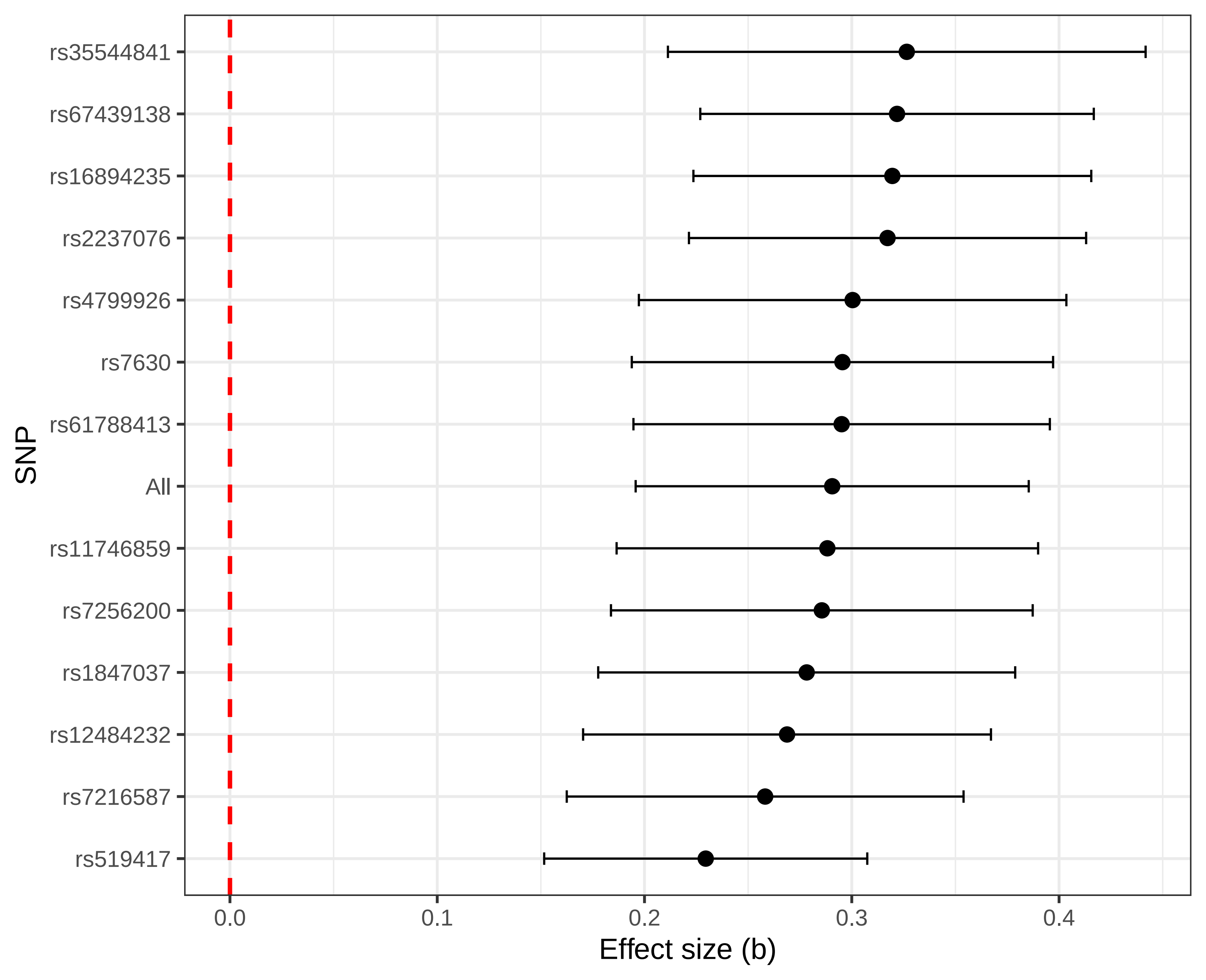

rs35544841
rs67439138
rs16894235
rs2237076
rs4799926
rs7630
rs61788413
All
rs11746859
rs7256200
rs1847037
rs12484232
rs7216587
rs519417
SNP
0.0
0.1
0.2
0.3
0.4
Effect size (b)
